# Development of intracardiac thrombus in a young patient with antiphospholipid syndrome while she was on rivaroxaban: Case report and literature review

**DOI:** 10.1002/ccr3.4137

**Published:** 2021-05-19

**Authors:** Elabbass A. Abdelmahmuod, Abdelrahman Hamad, Ahmed Almaghraby, Samah Mohamed, Maryam Alkuwari

**Affiliations:** ^1^ Department of Internal Medicine Hamad Medical Corporation Doha Qatar; ^2^ Department of Cardiology Noninvasive Labs Heart Hospital Hamad Medical Corporation Doha Qatar; ^3^ Department of Cardiology Clinical Imaging Hamad Medical Corporation Doha Qatar; ^4^ Department of Cardiology Cardiac Imaging Hamad Medical Corporation Doha Qatar

**Keywords:** antiphospholipid syndrome, APS, intracardiac thrombus, right atrium, rivaroxaban

## Abstract

The probability of right heart thrombus co‐existence should be considered in patients with antiphospholipid syndrome (APS) who have pulmonary and cardiac symptoms. The prevention and management of intracardiac thrombotic events include early use of anticoagulation therapy.

## Graphical abstract

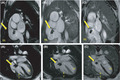

## 1 INTRODUCTION

Antiphospholipid syndrome (APS) is an autoimmune disorder characterized by acquired thrombophilia that leads to arterial/venous thrombosis. In patients with APS, stroke, transient ischemic attack, deep vein thrombosis, and pulmonary embolism are the most common thrombotic events. An intracardiac thrombus, a life‐threatening complication with a high risk of increased morbidity and mortality, can occur in patients with APS, but it is rare. Still, it is treatable by intensive anticoagulant treatment and or surgical removal. In APS patients, spontaneous intracardiac thrombus formation is a possibility. With a preference for the right ones, however, thrombus formation can occur in all cardiac chambers. On native valves or mural endocardium, thrombus may occur spontaneously or cause by manipulations such as catheter positioning or prosthesis valves. It remains uncertain about the mechanism of thrombus formation. Here we reported 24‐year‐old lady who presented to the emergency department with shortness of breath and was found to have an intracardiac thrombus in the right atrium. She was admitted to the hospital and received enoxaparin warfarin, which she improved and discharged. In conclusion, while unusual, the probability of right heart thrombus co‐existence should be considered in patients with APS who have pulmonary and cardiac symptoms. The prevention and management of repeated intracardiac thrombotic events include early use and maintenance of anticoagulation therapy.

Antiphospholipid syndrome is an autoimmune disorder commonly occurs in women characterized by autoantibodies directed against phospholipid on the plasma membrane.^1^

Antiphospholipid syndrome is usually associated with SLE but rarely can occur independently. APS is characterized by arterial/venous thrombosis and recurrent pregnancy loss.^2^

Antiphospholipid syndrome is a clinical syndrome diagnosed when meeting the following criteria one clinical and one laboratory. Clinical criteria that suggested APS include are ≥1 arterial/venous thromboembolism and pregnancy loss (stillbirth, intrauterine death, preeclampsia, premature birth, and fetal growth restriction).

Laboratory criteria that suggested APS include the presence of anti‐beta2 glycoprotein, positive lupus anticoagulant, and moderate‐to‐high anticardiolipin antibodies on at least two occasions 12 weeks apart.^3^

A potentially life‐threatening but treatable manifestation of APS is intracardiac thrombosis. Thrombus formation can cause embolic pulmonary and systemic events. Thrombi may be located in the chambers on the right or left of the heart. Some indicate that the right‐side chambers are preferred. Thrombus formation was reported more frequently on the right side of the heart in a study of 40 primary APS patients, contrary to valve involvement, which is usually on the left side.^4^

Typically, initial management includes anticoagulation, but if symptoms are severe, surgical removal of the thrombus needs to be done. Intracardiac thrombus needs to be differentiated from intracardiac myxoma.^5^

Here, we would like to shed light on the rare site of intracardiac thrombosis associated with APS.

## 2 CASE PRESENTATION

A 24‐year‐old lady with a history of provoked DVT and PE diagnosed after her car accident and kept under rivaroxaban, she lost her follow‐up, and she continued rivaroxaban for 18 months. She presented to the emergency department with shortness of breath, fatigability, and palpitation. On examination, she only had a malar rash; other examinations were unremarkable.

Her laboratory tests showed feature consistent with autoimmune hemolytic anemia see Table 1 (very low hemoglobin Hb 5.8, high LDH, low haptoglobin and elevated indirect bilirubin, and positive comb test).

**TABLE 1:** Showed feature of hemolytic anemia

Detail	Value w/units	Flags	Normal range
Bilirubin T	23.0 μmol/L	HI	0.0‐21.0
LDH	317 U/L	HI	135‐214
Haptoglobin	23 mg/dL	LOW	30‐200
Coomb test	Positive		
Hgb	5.8 gm/dL	CRIT	12.0‐15.0
Retic %	3.3%	HI	0.5‐2.5

Her other laboratory tests consistent with APS see Table 2 (low platelets 39, high APTT, positive lupus anticoagulant and cardiolipin, and low c3, c4).

**TABLE 2:** Showed features of antiphospholipid syndrome

Detail	Value w/units	Flags	Normal range
c3	0.77 gm/L	LOW	0.90‐1.80
C4	0.04 gm/L	LOW	0.10‐0.40
Anti B2 glycoprotein IgG Int	Positive	NA	
Lupus confirm	39.0 s	HI	27.7‐33.5
Lupus screen	105.3 s	HI	30.4‐45.3
Plt est	38 × 10^3^/μL	LOW	150‐400
APTT	84.2 s	HI	24.6‐31.2

Peripheral smear showed fragmented RBCs and spherocytes with thrombocytopenia.

Other autoimmune workups for SLE, rheumatoid arthritis, were negative.

**FIGURE 1:**
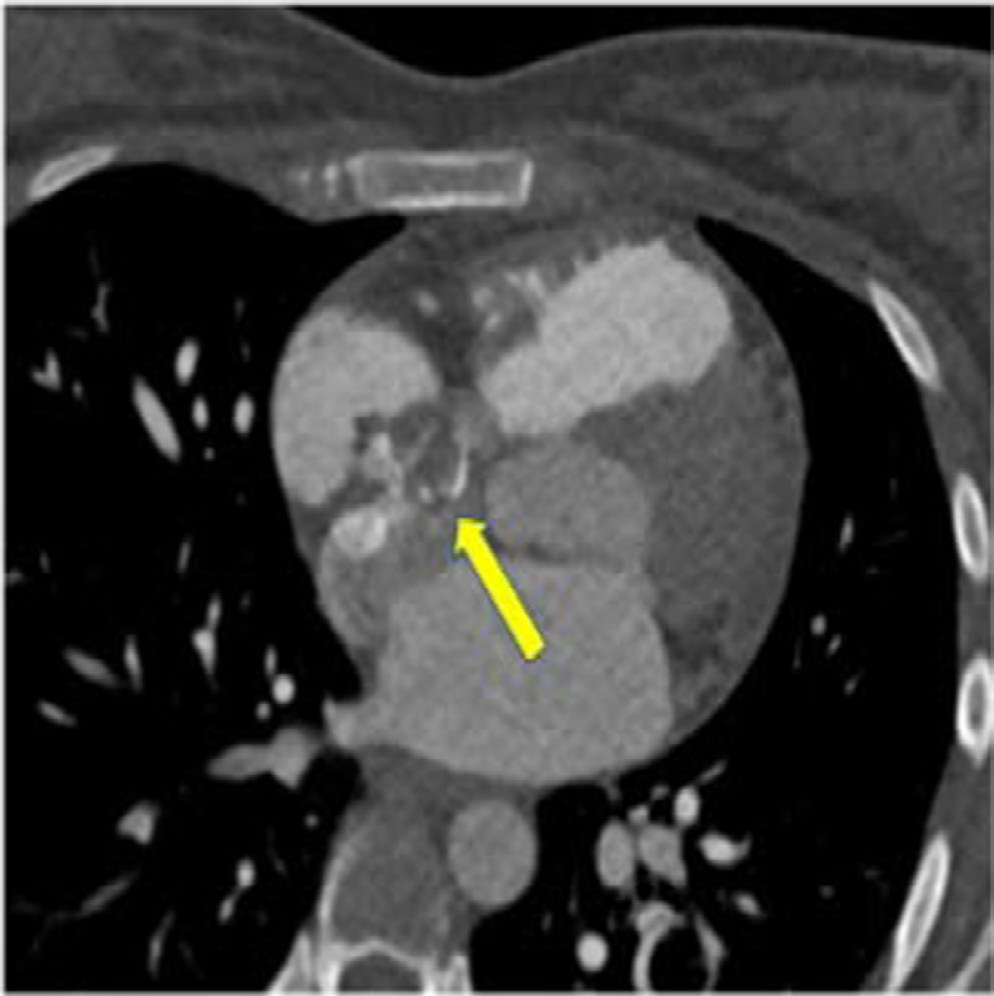
Axial CT pulmonary angiography: showing incidental right atrial filling defect (Yellow arrow)

**FIGURE 2:**
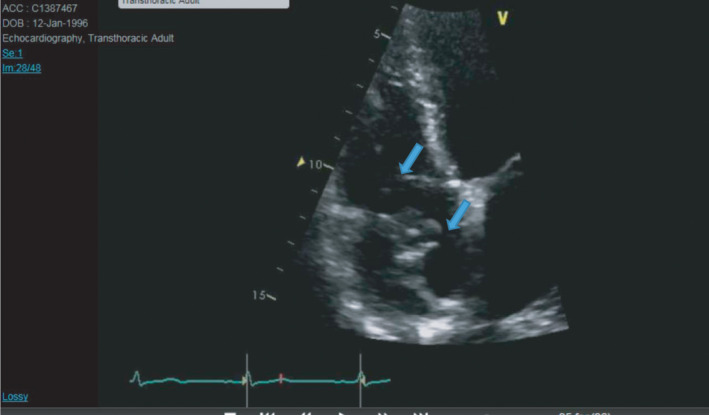
ECHO: showed mass in right atrium (Blue arrow) with suspicion of intra atrial thrombus

In the emergency department, CT pulmonary angiogram was done to rule out pulmonary embolism, which ruled out but showed incidental findings of right atrium defect (see Figure 1). Echo was done based on CT findings, and it showed a large mass seen attached to the right atrial wall, most probable with intracardiac thrombus (see Figure 2).

**FIGURE 3:**
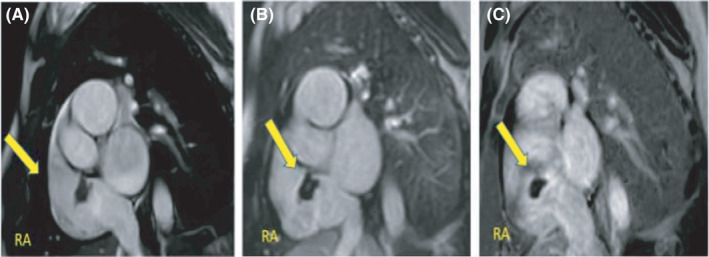
Cardiac MRI atrial short axis planes A, precontrast short axis SSFP CINE still image showing hypointense lobulated structure (yellow arrow) inferior to the SVC, and post gadolinium early B, and delayed enhancement C, short axis views showing no contrast uptake within the right atrial structure, consistent with atrial thrombus

**FIGURE 4:**
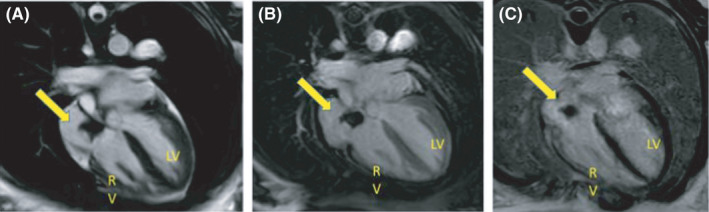
Cardiac MRI four chamber planes A, precontract 4 CH SSFP CINE still image showing a right atrial hypointense lobulated structure (yellow arrow), and post gadolinium early B, and delayed enhancement C, 4 CH views showing no contrast uptake within the right atrial structure, consistent with atrial thrombus

The cardiology team was involved and decided to do a cardiac MRI for confirmation instead of TEE because of her low platelets; MRI showed right atrial thrombus (see Figures 3 and 4).

She stopped rivaroxaban and started on enoxaparin (therapeutic dose 80 mg subcutaneous BID) and warfarin (5 mg oral daily) with a target INR 2‐3. She stayed for 5 days in the hospital with an improvement of her symptoms. She was discharged with regular follow‐up with the rheumatology clinic and showed that her symptoms improved on warfarin and repeated ECHO showed decreased the thrombus size. Her antiphospholipid antibodies were measured again after 12 weeks, which showed high titers.

## 3 DISCUSSION

Antiphospholipid syndrome is one of the most typical causes of acquired thrombophilia, leading to venous and arterial thrombosis. Deep vein thrombosis, pulmonary embolism, transient ischemic attack, and stroke are frequent thrombosis sites related to APS. However, the intracardiac thrombus is an infrequent site of APS‐related thrombosis.^6^

Diagnosis of APS is by one clinical and one laboratory, including arterial/venous thrombosis, recurrent miscarriages, and antiphospholipid antibodies (lupus anticoagulant, anticardiolipin, and beta2 glycoprotein) on two or more occasions at least 3 months separation.^7^

Intracardiac thrombus can be due to several causes of thrombophilia, which can be hereditary like factor v Leiden mutation, deficiency of antithrombin III, homocystinuria, or acquired causes like essential thrombocythemia and polycythemia vera. Antiphospholipid syndrome is a very uncommon cause of intracardiac thrombus.^8^

Cardiac manifestation of APS includes valvular dysfunction like Libman‐sack endocarditis, leading to mitral regurgitation or other rare valvular lesions such as aortic stenosis. Other cardiac manifestations of APS include coronary artery thrombosis with or without ventricular dysfunction.^9^

Our patient suffered from DVT and PE and missed diagnosis as provoked DVT secondary to immobilization followed road traffic accident. Although she missed her follow‐up and continued rivaroxaban for 18 months, she developed an intracardiac thrombus. This support the evidence that NOACs cannot prevent thrombosis associated with APS.

The underlying pathogenesis of thrombosis associated with APS is not understood; however, the procoagulant effect enhances thrombus formation.^9^ Intracardiac thrombus is unlikely but can occur on both right and left sides; however, it is most common on the right side and can be even leading to APS's diagnosis in the absence of other symptoms of APS.^10^

Intracardiac thrombi are frequently seen in patients with APS on the surface of prosthetic or morphologically irregular native heart valves. However, intracardiac thrombi tend to be rare on either structurally normal valve leaflets or unrelated cardiac valves and involving mural endomyocardial.^11^

Although antibodies are known to lead to the development of an intracardiac thrombus, the exact mechanism is still unknown. One of the suggested mechanisms is the disruption of physiological fibrinolysis by aPLs, which results in thrombus formation on the endocardial surface in the presence of predisposing factors. Other researchers have hypothesized that intracardiac blood flow pattern disorders may lead to thrombosis.^12^

Upon Literature review, few cases reported the association between APS and intracardiac thrombus. The first two reports were in young women presented with systemic symptoms without embolic presentation.^13^ In the absence of predisposing factors such as atrial fibrillation and signs of systemic or functional defects considered to encourage local stasis, primary APS patients are at high risk of developing intracardiac thrombus. Thrombi were found in 16% of primary APS patients by transesophageal echocardiography. It was observed more often in the heart's right chambers, and oddly, the thrombus was located in the left atrial appendage in one patient.^14^

One literature review summarized 10 intracardiac thrombi patients with APS. Most of these patients were women aged 30‐40 years. The right atrial chamber was the most frequent location of the intracardiac thrombus; however, thrombus was identified in the left atrium and left ventricle in some cases. One of these patients was a female teenager who had APS associated with systemic lupus erythematosus.^15^

In APS patients with intracardiac thrombosis, adequate maintenance anticoagulation therapy heparin and warfarin (target INR 3.0‐4.0) are required. The role of intervention in surgery remains controversial. The thrombus should be surgically removed as early as possible to avoid repeated intracardiac thrombotic events, and appropriate maintenance therapy with anticoagulation should be given. Treatment with warfarin anticoagulation along with cardiac surgeon consultation was recommended for management of APS‐related intracardiac thrombus. Up to date, for this unusual complication of APS, no randomized trials are comparing various treatment strategies.^16^

## 4 CONCLUSION

Intracardiac thrombus has rarely been reported in patients with APS. Such association should be considered because early diagnosis and proper management with anticoagulant therapy is the cornerstone for the prevention of preventable undesired lethal events.

## CONFLICT OF INTEREST

None declared.

## AUTHOR CONTRIBUTIONS

EAA and AH: wrote, edited, and finally approved the manuscript. AA, SM, and MA: involved in imaging review.

## ETHICAL APPROVAL

This case was approved by the Hamad Medical Corporation's Medical Research Center, and the patient consented to the publication of his case.

## Data Availability

Data and materials are available on reasonable request.
